# Supplementary material for: Identification of 3-Methoxyphenylacetic Acid as a Phytotoxin, Produced by Rhizoctonia solani AG-3 TB
Source: Molecules. 2023 Jan 12;28(2):790. doi: 10.3390/molecules28020790 (PMC9860971; doi:10.3390/molecules28020790)
Supplement: Supplementary file 1 [file molecules-28-00790-s001.zip › molecules-2109950-supplementary-done.pdf]

molecules

MDPI

Supplementary Materials

Identification of 3-Methoxyphenylacetic Acid as a Phytotoxin, Produced by *Rhizoctonia solani* AG-3 TB

Table S1. The the original data of inhibition rate of radicle elongation by toxin extraction

Test repetition	1	2	3	4	5	6	7	8	9	10	11	12	13	14	15	16	17	18	19	20	aver-age
Length of control radicle (cm)	2.46	1.74	1.82	1.91	1.87	1.99	2.72	2.84	2.97	1.76	1.91	1.62	2.48	1.89	1.94	2.97	1.79	1.96	2.85	1.95	2.172
Length of treated radicle (cm)	0.11	0.15	0.04	0.30	0.13	0.20	0.05	0.23	0.03	0.09	0.23	0.13	0.16	0.14	0.19	0.22	0.14	0.14	0.17	0.13	0.149
Inhibition rate of radicle elongation(%)	-	-	-	-	-	-	-	-	-	-	-	-	-	-	-	-	-	-	-	-	93.14

Table S2. The lesion diameter of bioactivity of each phase after extraction by activated carbon

Treatment	Lesion diameter (cm)	Significant difference	
		5%	1%
Precipitate	0	b	B
Aqueous phase	0.892 ± 0.165	a	A
Organic phase	0.042 ± 0.018	b	B

Table S3. Determination of biological activity of toxin components

Component	Lesion diameter (cm)	Significant difference	
		5%	1%
1	0	c	C
2	0	c	C
3	0.934 ± 0.236	a	A
4	0.051 ± 0.022	b	B

Table S4. Determination of bioactivity of each component after HPLC separation

Component	Lesion diameter (cm)	Significant difference	
		5%	1%
I	0.868 ± 0.198	a	A
II	0	c	C
III	0.0341 ± 0.034	b	B
IV	0	c	C
V	0.792 ± 0.273	a	A
VI	0.028 ± 0.035	b	B
VII	0.011 ± 0.072	b	B
CK	0.982 ± 0.233	a	A

Table S5. The elemental analysis of toxin component

Repeat	Percentage content (%)			
	C	H	O	N
1	65.01	6.16	28.32	0
2	65.01	6.18	28.38	0
Average value	65.005	6.17	28.35	0

Table S6. The hydrogen spectroscopy of toxin component

Chemical shift (ppm)	Proton number	Peak shape	Proton chemical shift (ppm)	Ascription	Remarks
15～11	1H	br	/	-COOH	
7.25	1H	m	6.87, 6.83	H5	
6.87	1H	d	7.25, 6.83	H2, H4, H6	J=7.3Hz
6.83	2H	m	7.25, 6.87		
3.80	3H	s	/	H9	
3.62	2H	s	/	H7	

Table S7. The carbon spectroscopy of toxin component

Chemical shift (ppm)	Ascription	Proton chemical shift（ppm）	Long-range proton chemical shift (ppm)
178.0	C8	/	3.62
159.8	C3	/	7.25, 3.80
134.7	C1	/	7.25, 3.62
129.8	C5	7.25	/
121.8	C2/6	6.87	6.83, 3.62
115.2	C2/6	6.83	6.87, 6.83, 3.62
113.0	C4	6.83	6.87
55.3	C9	3.80	/
41.2	C7	3.62	6.86, 6.83

Disclaimer/Publisher's Note: The statements, opinions and data contained in all publications are solely those of the individual author(s) and contributor(s) and not of MDPI and/or the editor(s). MDPI and/or the editor(s) disclaim responsibility for any injury to people or property resulting from any ideas, methods, instructions or products referred to in the content.
